# Supported Influence Mapping for Mobile Robot Pathfinding in Dynamic Indoor Environments

**DOI:** 10.3390/s24227240

**Published:** 2024-11-13

**Authors:** Paweł Stawarz, Dominik Ozog, Wojciech Łabuński

**Affiliations:** 1 Department of Computer and Control Engineering, Faculty of Electrical and Computer Engineering, Rzeszów University of Technology, 35-959 Rzeszow, Poland; p.stawarz@prz.edu.pl; 2 Department of Applied Mechanics and Robotics, Faculty of Mechanical Engineering and Aeronautics, Rzeszów University of Technology, 35-959 Rzeszow, Poland; w.labunski@prz.edu.pl

**Keywords:** artificial intelligence, decision support systems, influence mapping, mobile robots

## Abstract

Pathfinding is the process of finding the lowest cost route between a pair of points in space. The aforementioned cost can be based on time, distance, the number of required turns, and other individual or complex criteria. Pathfinding in dynamic environments is a complex issue, which has a long history of academic interest. An environment is considered dynamic when its topology may change in real time, often due to human interference. Influence mapping is a solution originating from the field of video games, which was previously used to solve similar problems in virtual environments, but achieved mixed results in real-life scenarios. The purpose of this study was to find whether the algorithm could be used in real indoor environments when combined with information collected by remote sensors.

## 1. Introduction

Pathfinding is a complex problem with the roots in the field of discrete mathematics, which has attracted much attention in recent times, due to the rise of demand for algorithms suitable for navigation of unmanned drones and autonomous vehicles. Pathfinding is the process of discovering the most suitable path between a given starting point and the destination. A path is an ordered set of vertices, where in the context of this study, each vertex corresponds to a point in a real-life environment. The possibilities and complexity vary depending on the type of the environment. In outdoor scenarios, the agent may utilize preplanned roads and pavements. Interior pathfinding differs due to the presence of an increased number of obstacles of different sizes. Indoor pathfinding is challenging as a subject, but viable solutions exist in the field of geographic information systems [1]. When dealing with autonomous vehicles, the agent must account for human interference, which further increases the complexity of the task. An environment in which such interference occurs is called a *dynamic* environment. Pathfinding in a dynamic indoor environment requires not only mapping the current state of the environment, but also keeping track of all the changes in real time. In the case of virtual environments, multiple solutions exist, often originating from a background related to video game design [2]. A different approach is assisted pathfinding, which is realized by propagating dynamically acquired information [3].

In this study, we combine both methods via a process called *influence mapping*, in order to establish whether it is a sufficient solution for pathfinding in simple dynamic indoor environments. We start by describing influence mapping, and the methods in which it can be used. Then, we present academic literature focused on the influence mapping in autonomous vehicles, with respect to limitations and effects. Next, we present our solution, which combines influence maps with remote sensors that acquire information dynamically. We then proceed by describing a testing environment that we have developed in order to test the solution. The paper ends with the description of our findings and suggestions for future research.

### 1.1. Influence Mapping

**Figure 1:**
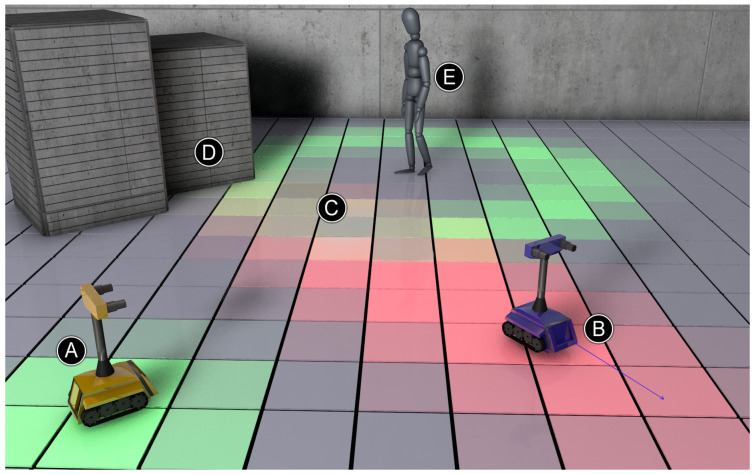
Visualization of an influence map with interesting elements marked. (**A**) Stationary positive influence source. (**B**) Negative influence source moving forward and leaving an influence trail. (**C**) Overlap of inverse influences yields a neutral zone. (**D**) Obstacles stop influencing propagation. (**E**) Non-point influence source.

Influence mapping is the act of assigning a value to a particular point in time and space. The value itself is referred to as *influence*. The task is performed by utilizing a structure called an *influence map* (often abbreviated to *IM*). An identical term can also be used to describe a diagram showing how one subject influences another, as in [4]. This paper focuses on influence maps, understood as data structures, and not data representation methods. In the simplest version, the influence map is a two-dimensional array of float numbers, as first seen in [5]. Figure 1 presents a visualization of an exemplary influence map. Modern approaches substitute the array with a more sophisticated structure, like a graph or navmesh, often depending on the structure used by the pathfinding algorithm. Basic concepts related to influence maps include influence spread/propagation, influence decay, granularity, refresh rate, and influence source parameters.

In the beginning, all of the cells/vertices of an influence map have a neutral value, which usually means zero, since there is zero influence on the map. Then, when an influence source appears, it starts projecting influence of a certain value, decided *a priori* by a designer or expert, and associated with the source type. A source that emits influence values that are above the neutral value is called a *positive influence source*, and sources that project values lower than the neutral threshold are called *negative influence sources*. For example, the agent running the program can project positive values of influence (as seen in Figure 1A), whereas other agents can project influence of a negative value, much like in Figure 1B. Other objects, entities, and events can project influences of different kinds and strengths. For example, in the case of an agent trying to stay at arm’s length from a human, people could be potential candidates for a positive influence source. The influence could be projected in a pattern around the source, and not strictly underneath the person, as portrayed in Figure 1E. This approach would maximize the positive value of cells at a distance from the person.

The influence spreads from cells/vertices associated with the source to other neighboring cells or vertices. The process is called *influence propagation* or *influence spread*, and can be seen in Figure 1A. The spread speed is defined by the spread function, which the system designer selects. Propagation means that when an influence source moves, it leaves behind a trail of influence. The effect can be observed in Figure 1B. The influence, if not constantly reapplied by an active source, will eventually fade, which is called *influence decay*, and much like influence propagation, it is controlled by the function chosen by the system designer. Decay means that a trail left by a moving source will slowly reach neutral values as the source moves away. Spread and decay can also lead to situations in which influences of opposite polarity combine into a neutral zone, as shown in Figure 1C. Walls and obstacles (Figure 1D) may change the way influence spreads, blocking it totally or suppressing the spread rate.

The 1969 article “A Model of Visual Organization for the Game of GO” [5] is the first use of influence maps in academic literature. The technique improved the virtual agent by making it aware of the board’s segmentation and the stones’ influence. The basics of influence mapping are covered in-depth in [6]. The author describes the idea and theoreticalally applicable examples and then proceeds to cover connected subjects, including parameter tweaking, accounting for terrain, and scalability. The chapter ends with a brief description of problems that arise from using influence maps in 3D environments, in case of which a navmesh solution is proposed over a standard grid representation. During GDC 2011, a whole hour-long session discussed influence maps [7], including basic theory, practical applications, tactical reasoning examples, and improving individual behavior. The panel also covered advanced subjects, including the usage of graphs or waypoint networks instead of grids and adjusting IM parameters. It is worth noting that the authors clearly stated that influence mapping is especially useful in two scenarios: in the case of *exciting terrain* and when there is a need for emergent behavior.

A chapter from the book *Game AI Pro 2* presents a memory-efficient, infinite-resolution influence map [8]. The solution eliminates the problem of data granularity but it is costly to query and update. In another chapter [9], a modular system allowing the combination of multiple map components in a variety of ways is presented. The advantage of this solution is the ease of creating and tuning a wide variety of specialized maps. During the Game Developers Conference 2015, the authors did a panel showing how to utilize both modular and infinite-resolution influence maps [10]. Influence maps were used to extract information about an area or to find a location with desirable traits. Each system layer was an atomic component, and the authors gave examples of proximity and threat maps based on a KD tree. The refresh rate was set to a value of one per second.

Other critical factors that need to be determined beforehand are the refresh rate and granularity of the map. Both of these parameters significantly impact the computational complexity, which in effect has side effects that may be unexpected to inexperienced users. Since both effects are triggered during the refresh cycle, the higher the refresh rate, the faster the spread and decay. Maps storing historical values may, thus, work better when refreshed less frequently. The larger the cells that form an influence map, the lower the computation time the map requires. Minor obstacles may be impossible to represent on large tiles. Similarly, if the tiles were be too small, most influence sources would occupy multiple tiles due to their overwhelmingly larger size.

### 1.2. Potential Fields

Artificial potential fields (often abbreviated to *PF*) are a different method of combining influence with spatial–temporal context. In a potential field, each cell, instead of storing a singular influence value, stores a vector describing the direction towards a cell with higher (or lower, depending on the implementation) influence. Artificial potential fields are often devoid of influence propagation and decay, offering more direct control over each cell’s value. The areas applying potential fields range from fluid dynamics [11] to autonomous ground vehicles [12], with reviews available in open access.

Despite the differences, both techniques are virtually interchangeable in the context of pure representation. Each influence map can be transformed into a potential field by comparing values on a cell-by-cell basis, but the exact value is lost. Similarly, a potential field can be converted into an IM by assuming a base value in a cell and increasing/decreasing it by a set amount in neighboring cells, depending on the direction of the vector. After that, the IM can be normalized to a suitable range. While conversion may seem redundant, many issues that are solved by influence maps remain problematic when approached with PF-based solutions. For example, the authors of [13] attempted to evenly position multiple robots on a circle and used two potential fields to solve the problem. On the other hand, a solution using a singular influence map was presented eleven years earlier in [14] in the role of a trivial example. The cases presented in both sources are obviously different, the first being a real-world problem and the second a simulation in a virtual environment.

This suggests that potential fields are preferable in robotics, whereas influence maps are more common in virtual environments, like video games or simulations. The ease of implementation can explain the correlation between use cases and algorithm types. For example, since two-dimensional arrays of numbers are standard in most programming languages, IMs are a go-to method in virtual environments. On the other hand, it is non-trivial to extract precise influence values from a natural environment due to sensor limitations, thus making PFs more suitable for such applications.

### 1.3. Advantages of Influence Mapping Techniques

Mobile robot pathfinding can be defined as a process of designing robot motion from point A to point B over the shortest distance, in the least amount of time and with collision-free movement. On the basis of the literature, two subsets of algorithms can be distinguished: global pathfinding and local pathfinding. Global pathfinding depends on the knowledge of the environment and methods of generating robot’s route beforehand. This is also known as offline or static planning. On the other hand, in local pathfinding algorithms and methods, the robot is partially or completely unaware of its surroundings, and it must be equipped with sensors to monitor it in real time. This is also called online or dynamic planning [15]. The influence mapping can be considered a fusion of both the approaches defined above, which are able to work synergically for finding paths and move along them. It can also be used as a tactical planner, as was shown in [16]. The paper describes the research on multivehicle tactical planning, pathfinding, collision avoidance, trajectory generation, and dynamic vehicle control. The authors used the influence map as a low-level path planner. To improve the results, the data were filtered in spatial and time dimensions. The next step was to feed the computed map to a high-level path planner. Each mobile robot used the map to generate a path with the A* global pathfinding algorithm. The results obtained in this research show that using influence mapping combined with standard methods enables efficient tactical planning and generates safe paths for multiple robots with limited information about the environment. Moreover, expanding pathfinding algorithms with influence maps leads to enhanced situational and environmental awareness of robot systems, both globally and locally. Providing additional information in the form of weighted values improves the time and speed of processing, both in static and dynamic environments. Multirobot systems with the entire range of sensors can monitor moving or changing objects and their presence can be projected on the influence map, contributing to the overall performance and accuracy of the map. As proven in other work, using influence maps in multirobot systems can result in synergically enhanced performance of said system and improvement of the quality of the pathfinding process [17].

## 2. Influence Maps for Mobile Robots

Robots can be divided in many different ways. One of them is based on the design of the robot and, consequently, on how it can move. The broadest distinction can be made into mobile robots [18,19,20,21,22] and robotic arms [23]—the latter ones are most commonly associated with industrial applications in production lines for machining [24,25], collaboration with humans [26], or palletizing [27]. In most cases involving mobile robotics, it is important to ensure that robots can effectively navigate a workspace that contains both free areas and obstacles. The task is crucial, as collisions increase the cost of movement and, in the case of industrial robots, can lead to the destruction of the tool or a workpiece. The programming of mobile robots can be implemented using the potential field algorithm or an influence map, which is a broadening of the advanced cost map that takes into account not only the obstacles themselves, but also additional features, such as the robot’s energy level, the distance to the target, distance to potential obstacles, and others. In robotics research, the term “influence map” is used rarely. Rather than providing detailed instructions for next steps, advanced robot control theory and its applications involve setting goals and then pursuing them while minimizing errors or costs. This can be achieved using well-known pathfinding techniques, e.g., graph search [22]. For this purpose, an influence map can be compared to the evaluation function in computer games, which determines the value of a given game state. Adding an influence map positively impacts the ability to visualize navigational data.

### 2.1. Strategy and Tactics

In 2003, Zigoris et al. used influence maps to store information about the position and velocity of every object sensed by the robot [28]. These data were then used in a multiagent real-life game called RoboFlag. In 2005, a paper titled “Digital Pheromones for Coordination of Unmanned Vehicles“ proposed the use of digital pheromones to control uncrewed aerial vehicles [29]. The paper introduces many novel elements, including ghost agents and pheromone vocabularies. The proposed approach resulted in a decrease in randomness and helped to understand better the emergent behaviour. During the 11th International Conference on Control, Automation, Robotics, and Vision, Atyabi et al. presented *MAGICian*, a simulator designed to be a test-bed for heterogeneous teams of uncrewed robots [30]. Both local and global influence maps were available, and teams working with the simulator could use them for exploration, coordination, and planning. However, out of all the participants during the competition, only the creators of MAGICian used an influence map. Their entry was called WAMbot. Other teams achieved better results [31]. In [17], WAMbot is described in detail, with an in-depth analysis of the reasons behind the mixed performance. In 2012, a subset of the authors from the MAGICian competition researched a multilevel hierarchical motion planning system [16]. The solution combined influence mapping, A*, Elastic Bands, and Dynamic Window Approach. The data stored in the influence map were a combination of the output from an exploration algorithm and waypoints manually set by humans. Frey and Shulte also worked on cooperation between crewed and uncrewed vehicles, but in a different scenario, in which UAVs would assist a helicopter [32]. Enemies were represented as *influence points*, with strength assessed based on the information in military databases. The approach does not use an influence map but proposes it as a future research topic as a part of a tactical situation analyzer. Ref. [33] focused on bot development for military simulators. The agent described in this paper makes use of a threat map, which is used for pathfinding and enemy avoidance. In 2021, Dubey used influence maps and potential fields combined with genetic algorithms in their thesis [34]. Various scenarios, both in the context of steering UAVs and strategy games, are provided therein.

### 2.2. Navigation

The two main applications of influence maps in mobile robotics found in the available literature are pathfinding and environment mapping. In the papers [17,35], a system is described that participated in The Multi Autonomous Ground-robotic International Challenge (MAGIC) 2010. The goal of the participants was to build a mobile robot system that would meet certain predetermined criteria and perform tasks in the context of defense systems. The authors proposed a navigation system that allowed robots to move efficiently between target points, ensuring that they avoid collisions with obstacles, other robots, and various hostile objects. Movement planning took place at four levels: Exploration, High Level, Medium Level, and Trajectory Planning. The high-level planner managed the tactical navigation of the robot fleet using influence mapping techniques, ensuring that robots did not approach enemy units. In this case, the influence map gathered a variety of tactical information that aids the navigation system in planning optimal movement. In the classic approach to influence mapping, enemy facilities are assigned with negative weights, while allied units receive positive ones. To improve this approach, influence map data are filtered in spatial and temporal dimensions to ensure knowledge of the current tactical situation. In addition to the influence map, a tension and vulnerability map were also generated. The proposed approach allowed the identification of areas of conflict and safe zones, which enabled robots to move while neutralizing threats or protecting selected units or areas. In the paper [36], a novel approach to map evaluation is presented and it uses artificial objects placed in the environment, called *reference markers*. By using known reference points and the positions of reference markers identified on the map, it is possible to assign a number of qualitative features for a given map. These features are weighted to calculate a final score, dependent on the application domain. The proposed approach was evaluated during the 2010 NIST Response Robot Evaluation Exercise. A method for detecting and localizing unusual activity in crowded environments around a robot using an autoencoder based on deep learning was described in [37]. The method uses the motion influence map and spatial–temporal features obtained from the autoencoder for classification, which achieves comparable accuracy to modern techniques on standard datasets.

### 2.3. Image Processing

Another area where influence maps can be applied is the image processing domain, specifically color transfer and correction. One of the possible applications where the influence maps are used is a method for correcting the color of an object in the target image, which was presented and analyzed in [38,39,40,41]. The main advantage of this approach is that the user does not have to select an object by its shape, which can be difficult for complex or small areas. It is selected in terms of its range and color statistics. The source color can be specified as a single color or as an area of another image, and after collecting information about the target color range, a Color Influence Map (CIM) of the target image is prepared. This is a mask that determines which parts of the target image will be influenced according to the selected color range. The next step in this algorithm is the color change process itself, which is performed on the target area according to the prepared CIM. CIM contains color transformation weights for each pixel of the target image. Each pixel’s weight is determined by its proximity to the color range selected by the user and stored in the color statistics information. This is the Mahalanobis distance between the pixel and the center of the color distribution, determined by the stored color statistics values. Instead of the Mahalanobis norm, the Euclidean norm can be used, as has been shown in [42]. The work [43] presents a method that allows color correction of multiple photographs automatically and simultaneously, even without knowing the original state of the image. With the ability to recover correct color and spatial information from images, more precise color correction and many other image restoration tasks can be performed—including filtering and denoising, blur reduction, and transformation of images from grayscale to other color spaces, for example RGB. The paper [44] proposes a video coding scheme based on the spatial–temporal influence map PVC-STIM (*perceptual video coding—spatial–temporal influence map*). The research was divided into two stages. First, a new perceptual model was prepared, taking into account many perceptual features of the human visual system (HVS), with a special focus on the combination of several spatial and temporal features, i.e., spatial masking effect, spatial stimuli, visual clarity, and temporal movement attention. In the second step, the proposed perceptual model is incorporated into classical video coding to adjust the Lagrange multiplier to assign visual quality, thereby improving speed, performance, and perceived distortion. Preparing the described framework, both spatial and temporal influence maps were incorporated and applied.

### 2.4. Anomaly Detection

Influence maps are also used in a few different practical applications in a variety of fields at the intersection of image processing, artificial intelligence, and robotics. One example of such an application is the monitoring of human movement to classify unusual behavior that could pose a threat in crowded rooms or at mass events, as in [45]. The paper presents a method for detecting and finding unusual activity in crowded areas. The developed algorithm utilized a large number of surveillance cameras to collect and analyze video and image streams. For this purpose, deep neural networks based on an autoencoder were used to conduct the process of categorizing anomalies. In this application, the Optical Flow is calculated using a motion influence map and passed to the neural network. This way, the spatial–temporal features obtained from the output of the encoder, are used for classification. The k-means method is then used to classify the extracted features. Experiments were conducted on standard crowd datasets; on the basis of the obtained results, it was noted that the proposed model achieves comparable accuracy to state-of-the-art techniques. The authors of [46] also developed a method for detecting unusual human activity in crowded areas. Instead of focusing on detection or segmentation of specific people in individual frames/images, the authors developed a motion impact map to represent human activity. A key element of this map is the effective representation of motion features, such as speed, direction of movement, and the size of objects and their interactions in a sequence of frames. With this map, a universal algorithm was prepared to detect both global and local abnormal activity. The study in paper [47] focused on detecting suspicious behavior in public places using a video surveillance system. The study involved developing a system that automatically recognizes suspicious activity using computer vision technology. It used the OpenCV library to classify various behaviors in real time and a traffic influence map to analyze and represent the changing traffic. In a similar way to human behavior and movement, animals can also be monitored. An example is the research in the work of [48], where a method based on a modified motion influence map combined with a recurrent neural network (RNN) was used to accurately monitor local abnormal behavior of schools of fish in intensive aquaculture. The method was designed to systematically detect, locate, and recognize local abnormal behavior of tilapia fish. First, the movement features of the entire fish stock were extracted using a particle advection scheme, without foreground tracking and segmentation. Then, based on this, a movement influence map was constructed, representing the characteristics of interactions within the fish stock, considering factors such as speed, direction, distance, and visibility. Then, based on the constructed motion influence map and using a properly prepared RNN, the detection and localization of local abnormal behavior was conducted. The approach to analyzing the types of traffic of groups of objects can also be applied to the study of traffic management. In paper [49], the process of monitoring logistics maps using GSM/GPS communication system was analyzed. An influence map was used to optimize travel routes using GPS data. According to the authors, this approach can be used to design new roads, minimize traffic congestion, increase safety in specific areas, plan the most efficient logistic routes, and select the location of police checkpoints.

## 3. Experiments

An influence map may be useful in applications where robots move through a diverse environment with a large number of obstacles or when adaptive behavior is required due to the presence of other moving entities. An important factor in favor of using this algorithm may be the presence of paths with different traffic capacities and the presence of bottlenecks. To test whether influence mapping can be combined with data from remote sensors safely, without risking damage to people or equipment, we created an artificial physical environment, in which humans were responsible for introducing the most significant changes that affected the occurrence of obstacles along the robot’s route.

### 3.1. Environment

Our first goal was to design an environment that would be as close as possible to a real-life scenario, while maintaining control over its elements. Our target scenario was a small warehouse with an inner product pick-up point and multiple entrances from which a semi-steady flow of workers would move toward the central point. This setup incorporates all the hazardous traits of a common indoor environment, which an autonomous robot is required to traverse. When designing the experiment, we focused on fulfilling a list of requirements. First, and foremost, the environment was required to have simple topology limited only by straight walls and right angles. Secondly, it had to contain multiple spatial–temporal obstacles. Lastly, it had to be an environment in which people could freely move. By designing the testing environment with regard to the aforementioned requirements, we could ensure it was fully controlled, which meant all the participants were safe during tests, and that the risk of damaging equipment was low. We also had full freedom over the placement of remote sensors.

**Figure 2:**
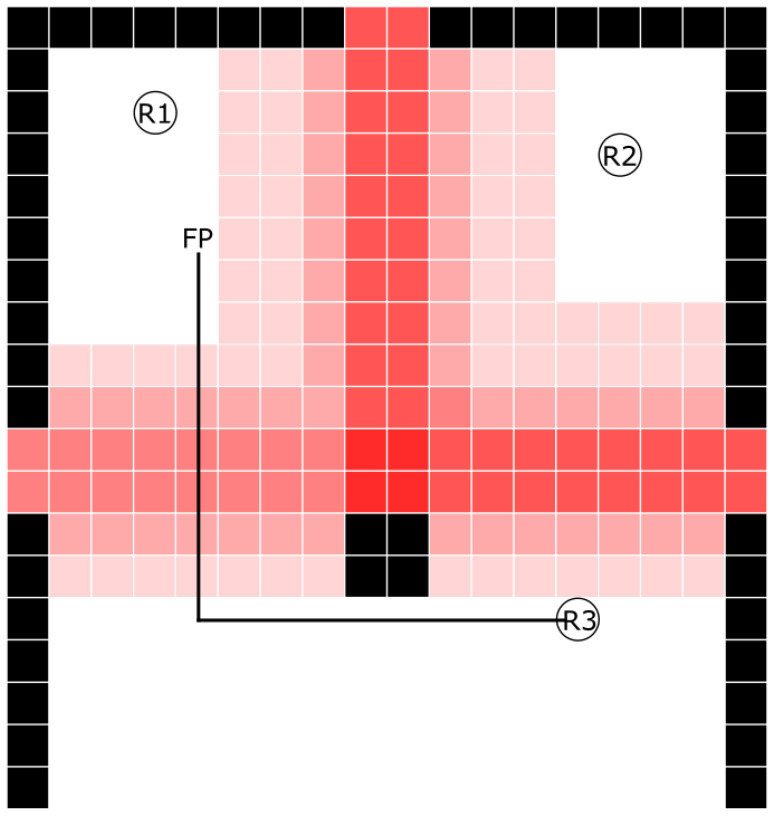
Diagram of the operating environment, including an influence map with the planned robot trajectory marked.

The final iteration of our testing environment, with regards to resources we were able to allocate during the research, was a single room with three entrances, one at the top, and two on the sides (Figure 2).

Furthermore, in the middle of the room, there was a pick-up point, which is considered a permanent obstacle in the problem under consideration. Participants generating human traffic (moving obstacles) were asked to enter the room through one door, interact with the center point, and leave the room through another door. There were no time limits regarding their traverse time, and no predefined order of entry or exit points. The environment was fully mapped by three LiDARs preceding the main testing phase. The result of the mapping was a virtual representation of the environment, including all fixed obstacles—the walls and the pick-up point in the center of the room. Those kinds of obstacles are marked with a black color in Figure 2. Apart from stationary obstacles and moving participants, there were three other robots present in the room. The robots are denoted by symbols R1, R2, and R3 in Figure 2. In the second experiment, the additional robots were responsible for scanning the environment and transmitting information about the detected obstacles to the central unit, which was responsible for implementing the influence map algorithm.

### 3.2. Influence Mapping

In the study, an influence map was used to solve the problem of finding the most optimal route for the robot. The environment in which the robot moved consisted not only of fixed obstacles and other mobile robots, but also involved the movement of people. Humans were responsible for introducing the most significant changes, in particular, affecting the occurrence of obstacles along the robot’s route.

In the software, the robot’s environment is represented as a graph that is a regular set of nodes, equidistant from each other. Every node could have a maximum of eight edges if it was possible to move from it to any of the neighboring vertices. If there were solid obstacles in the real environment, the nodes representing the workspace and adjacent to such an obstacle had a correspondingly smaller number of edges.

The algorithm generating the influence map, the most important part of which is shown in Listing 1, first made a copy of the current influence map. All changes were made in the copy, but all the values were extracted from the original version. This way, the situations where a change in the influence value in a given node affects the result of applying the same value to neighboring nodes in the same iteration have been eliminated.

Listing 1 **Fragment of the function generating the inflow map written in Matlab.**

Decay = 2;

Momentum = 120;

CopyMap = InflenceMap ( MainMap );

for node = MainMap . Graph

node . value = Calculate_Influence (‘expotential’, Decay

  , CopyMap . Distance ( node .id));

end

MainMap . UpdateGraph ( Enviroment . CurrentObstacles ,

  Momentum );

First, the algorithm determines the influence value for each node. Its composition is influenced by the distance from other nodes and their value computed in the previous iteration, as well as the influence decline function, which takes an array of decay parameters responsible for the parameter values of this mathematical function. In the case of very trivial tasks, it can be assumed that the influence decay function is linear. However, non-linear functions may provide better results, depending on the needs of the creators. Due to the low density of graphs and the systematic traffic of users, the exponential function has proven to yield the best results and was used in the final solution.

The result is then compared with the current reading of the position of moving obstacles. The data are passed through the *CurrentObstacles* array, where each value is assigned to a corresponding node. The *Momentum* parameter is responsible for the speed of changes, which in effect determines how long a given event must be remembered by the influence map. The value of this coefficient depends on the pace of changes taking place in the real environment.

### 3.3. The Problem of Signal Occlusion

We attempted implementing the idea described in Section 3.2 without the use of remote sensors. However, the resulting influence map contained errors, which we were unable to correct programmatically. The errors themselves were mostly invalid or missing the influence values originating from moving obstacles. We then traced all the problems to one source: *signal occlusion*. Even in an environment as spatially simple as in our experiment, the central column still blocked the mobile robot’s vision. A solution to this problem would be the addition of different kinds of sensors. However, that is not always possible, either due to technical limitations or economic costs. Moreover, parsing data from multiple sensors in real time is a computationally expensive task. We decided to test a different approach, which was to utilize remote sensors already present in the environment. In our case, other multiple mobile robots were present, all of which were equipped with LiDAR technology. This solution offers many advantages over the single multisensory robot solution. Each robot can be potentially identical, and parse data in the same computationally inexpensive way. Moreover, the environment itself does not have to be modified, as no other sensors are required. The only potential downfall is the fact, that even with multiple robots, their vision can overlap. In this paper, we tested how impactful the problem of vision overlapping is, and whether utilizing multiple mobile robots with singular sensors can be a potential solution to signal occlusion and enable influence mapping techniques.

### 3.4. LiDARs and the Environment

To recognize obstacles in the environment, three RPLIDAR S2 LiDARs (Shanghai Slamtec Co., Ltd., Shanghai, China) were used. Their maximum obstacle detection radius is at least 10 m (for objects that poorly reflect laser light, e.g., black, matte surfaces) and up to 30 m (for objects that reflect well, e.g., white, shiny surfaces). The laser uses a light source with a length of about 905 nm (Infrared Light Band). During the tests, the LiDAR rotation speed was set to 10 kHz (600 rpm). It allowed for measuring the distance to obstacles with an angular accuracy of 0.12 degrees—3000 measurements per one LiDAR rotation. These data were sent via the UART protocol to the robot microcontroller and then to the server.

**Figure 3:**
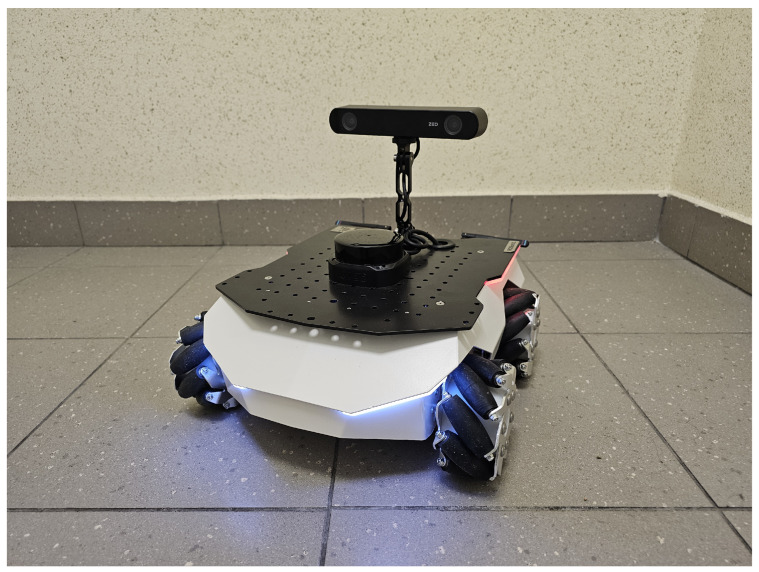
One of the robots used during the research.

The LiDARs were placed on robots (Figure 3). This solution results from a design often used in industrial mobile robots, in which LiDARs are used to scan the environment, avoid obstacles, and recognize the position of robots. In addition, the movement of the robots allows for updating the influence map in all areas used by the robots without the need to use many stationary sensors throughout the entire work area.

In the case of the study described, and placing the LiDARs on the R1 and R2 robots, as shown in Figure 3, the two LiDARs covered the entire area where there were moving obstacles, together with their area of operation. Hence, there was no need to move them around the area where the research was carried out. It would be enough to use only one LiDAR to monitor the entire space relevant to the research, but the algorithm described below would not test the construction of the influence map based on data from many independent sensors, which is an important part of enabling its applications in real industrial environments. Hence, the decision was made to use also a LiDAR on the R3 robot moving during the research. In a real-life scenario, where the LiDARs need to cover a larger and more complex space, the positioning of remote sensors could be determined by a dynamic coverage algorithm [50].

**Figure 4:**
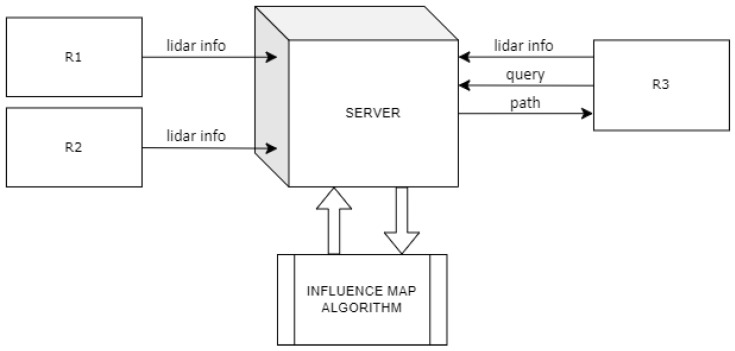
Communication flowchart in the study.

Each of the robots participating in the study (R1, R2, and R3 in Figure 4) sent data recorded by the LiDARs mounted on them to the server at a frequency of 2 Hz. The server processed the data, and divided obstacles into static obstacles and dynamic obstacles, depending on their movement. Additionally, preprocessing allowed the presence of robots to be ignored, so that they were not considered a permanent disturbance on the influence map. The obstacle data were then used in the algorithm that generates the influence map. The generation of the influence map was done by the server CPU (Intel i7-7700 (Intel Corporation, Santa Clara, CA, USA)), and took less than 50 ms. The algorithm was executed in a single thread as a Matlab script. Such a short execution time would allow an increase in the frequency of obstacle analysis, but it would not affect the conducted research. This was confirmed by experiments performed at a frequency of 1 Hz, where very similar data were obtained. However, it should be noted that for future applications in larger indoor spaces, there is a possibility of optimizing the algorithm execution time.

As shown in the communication diagram (Figure 4), the R3 robot could send a query to the server with a specific location (node id), which it wanted to reach. In this case, the server executed the Dijkstra algorithm using the values of the influence map to find the most optimal path. The R3 robot received a response from the server in the form of a list of nodes leading to the destination.

## 4. Experiments

Figure 2 shows a graphical interpretation of the influence map generated by the above algorithm. This map contains a fragment of an exemplary room, where the operation of the algorithm was checked. The nodes responsible for fixed obstacles (recorded by LiDAR before testing with moving obstacles) are marked in black—these are the walls and the pole in the center of the room. The image shows the lower part of the room, where no movement of other obstacles was generated during the test. There were three robots in the room. They are marked with symbols R1, R2, and R3. All robots were responsible for scanning the environment and transmitting information about the detected obstacles to the central unit, which was responsible for implementing the influence map algorithm. Husarion ROSbotXL robots were used for this purpose. Each of them had a RPLIDAR S2 LiDAR (Shanghai Slamtec Co., Ltd., Shanghai, China). These LiDARs were used to scan the robots’ surroundings and collect information about emerging obstacles. If an obstacle was detected, it was assigned to a place on the graph that corresponded to the physical area of the room. Information about an obstacle in a given area was sent to the central unit. The information was then stored in a table called *CurrentObstacles*, which is used by the influence map algorithm. The result of the algorithm determining the influence map is presented graphically in Figure 2 by the intensity of the red color. The graph fields for which the influence value is equal to 0 are white. For influence values greater than zero, the intensity of the red color is proportional to the influence value.

There were three entrances to the room, visible in the diagram (Figure 2) at the top and on the left and right. Furthermore, in the middle of the room there was a pick-up point, which is considered a permanent obstacle in the problem under consideration. Users generating human traffic (moving obstacles) were asked to enter the room through one door, interact with the central point, and leave the room through another door. As can be seen in Figure 2, the highest influence values occurred (the most intense red color) on the route connecting the door to the room and the central point. Due to the structure of the building, most pedestrian traffic was generated between the upper entrance and the one on the right side of the room. This fact is reflected in higher influence values—a more intense red color than in the case of the path from the middle to the door on the left side of the image. Additionally, the exponential function used as the function of distance generated a lower influence value in nodes adjacent to user paths, which is also presented in Figure 2 in the form of desaturated red fields.

The task carried out as part of the study was to determine the route for the R3 robot from its current location (marked in Figure 2) to the final location *FP*. This task took into account signals recorded by using distance sensors in the form of LiDARs located on each robot. The use of data from the sensor network allowed for reliable implementation of an influence map in the work environment of a robot swarm. Through the use of an influence map, these data influenced the results of the decision-making algorithms responsible for determining the optimal robot route. In a situation where the user’s influence on the robot’s route was not taken into account, the algorithms used to determine the best path picked the shortest possible route. It ran diagonally, bypassing the central obstacle on its right and upper side. A different route was picked when the influence map was used. The goal function took into account both the length of the route and the sum of influence, prioritizing the latter one. The route with the minimal value of the goal function was considered optimal. This approach resulted in the lowest probability of collision with a moving obstacle (person) but would extend the actual travel time. The algorithms for determining the most optimal route began to return the result marked in Figure 2 with a black line. This route ran along the lower and left side of the central obstacle. Even though it was physically longer than previously determined, the algorithms correctly took into account the time required to overcome it, using the data collected by the remote sensors.

**Figure 5:**
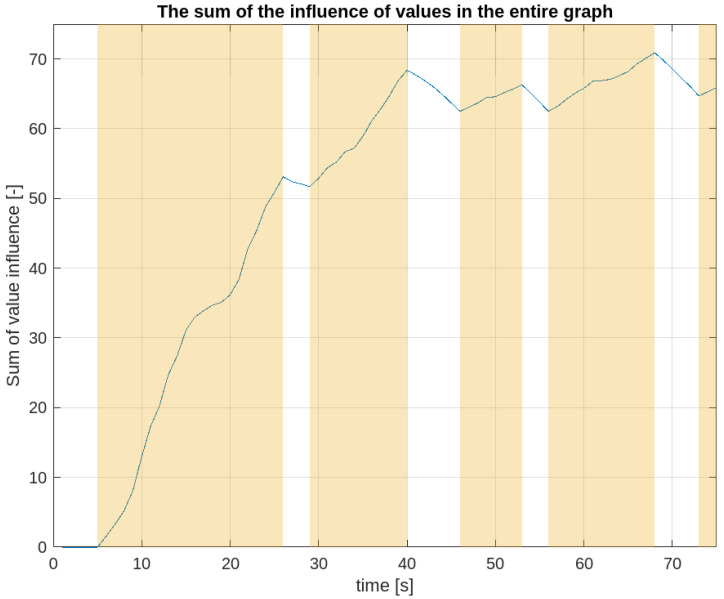
The sum of the influence of values in the entire graph.

Figure 5 presents a graph that visually presents the sum of influence values during the first 75 s of an exemplary experiment, which was considered the most representative among the 10 conducted. The room in which the research was carried out to determine the influence map was about 8 m wide. Each of the nodes of the graph representing the influence map shown in Figure 2 corresponded to the area of a square with a side length of 50 cm. If the algorithm detected an obstacle in a given area (excluding those places where a robot reported its presence or which were classified as permanent obstacles), it triggered a change of the influence value in a given node of the graph and the neighboring nodes. The ordinate axis of the graph (Figure 5) shows the sum of the influence values from all nodes of the graph. As can be read from them, for the first 5 s of the experiment, no obstacle was detected, and hence, the influence values in all nodes are equal to zero. From the 6th second, we observe an increase in the total influence value, which corresponds to the presence of the first human during the experiment. The total value growth lasts until 26th second, which was casued by the presence of a participant in the range of the LiDARs placed on the robots. In addition, a decrease in the growth speed can be observed between the 15th and the 20th second. This corresponds to the period when the person approached the pick-up point placed in the center of the experiment area and took an item from the shelf located on this point. The period of time when this and other people were in the LiDAR range is marked in Figure 5 by the pale yellow background.

From the 26th to the 29th second we can observe a decrease in the total value, which results from the influence value decay in individual nodes with time, while no obstacles are detected in them. Then, from the 26th second, another person entered the room. It was not until about the 33rd–34th second that they reached the pick-up point. At this time, we can see an increase in value, which is caused by an increase in the value of influence in nodes between the upper entrance and the pick-up point, while maintaining the values in nodes between the pick-up point and the right exit. The latter values resulted from the movement of the first person, and gradually decreased, but still had a high value. Then, the second person headed for the exit in the opposite direction as the first person. This caused an increase in the influence value in new nodes, which until now had a value of 0, and at the same time, the nodes towards the exit chosen by the first person still had positive values, which dropped slightly. Hence, significance in the total value generated by the activity of the second person occurred. The drop in the value on the graph for the next 6 s indicates the absence of people in the tested area at that time; then, it increases from the 47th second as a result of the third person quickly passing through the room. From the 57th second, two people entered the research area one after the other and went to the exits on the opposite sides. This resulted in an increase in the value of the influence in all nodes on the passage route. This caused a record increase in the total value. Then, on the graph, we observe a several-second drop in the value of the influence caused by the lack of people in the research area. The presented fragment of data ends after the next person enters the room. Already at this stage, it can be seen that depending on the intensity of entries into the room by individual people, the value of influence stabilizes at a certain level.

The experiments were performed multiple times, with different values of variables. The experiments carried out lasted about 5 min each and included the passages of 15–20 people. The limitation of the data presented in Figure 5 to 75 s is due to the need to maintain the readability of the graph and at the same time allows for the presentation of the characteristics of the described algorithm and the data returned by it. The saturation of the sum of influence happened in each experiment, but at a slightly different time frame, which was heavily influenced by the ratio between the influence propagation speed and the influence decay speed.

In order to determine the path with the shortest travel time of the robot, it was important to determine two coefficients. The first one is the travel time coefficient depending on the value of the influence in a given node. In the discussed case, a multiplier of 4 was assumed. This means that the travel time through the node with the highest possible activity was four times longer than through the node without the presence of dynamic obstacles. The second parameter is momentum presented in Listing 1, which was responsible for the speed at which the value of the influence decreased in individual nodes when no obstacle appeared in them. In the discussed case, it had a value of 120, which means that this value decreased to zero within 2 min. Additionally, in the position of the robot and its destination, which was presented in Figure 2, the choice of the exit to which the people headed during the experiment is important. If the exit on the left side was chosen at the beginning of the study, the robot was always instructed to follow the shortest possible route of about 6.4 m to the target, avoiding the pick-up point from the right, because then it blocked only the path from the entrance at the top to the middle of the room, increasing the travel time. A different route was picked in a situation when the participants preferred to leave through the door on the right side. In that case, the longer route (marked in Figure 2), which measured about 9 m, turned out to be more advantageous, because it intersected only one path with higher influence values. Our research indicates that this behavior was caused by the fact, that the time needed to cover two paths with increased influence values was added to the previously shortest route, meaning it became virtually longer.

## 5. Conclusions

This paper presents a case study of combining influence mapping techniques with remote sensors in solving mobile robot pathfinding problems. The research focused on scenarios in which multiple human and artificial moving obstacles were present in the environment. The environment itself mimicked the interior of a small warehouse with three entrances and a pick-up point in the middle of the room. A solution utilizing three LiDAR sensors was tested and proved viable in the aforementioned environment. Two LiDAR sensors were remote, which enabled the production of a holistic interpretation of the movement performed in the room, and converting it into an influence map. The influence map was then used by a mobile robot to plan a path that is short and does not collide with static or dynamic obstacles, even when supported by a small number of remote sensors. The results prove that combining influence mapping techniques with remote sensors is a potential solution to mobile robot pathfinding in dynamic multiagent and multiperson interior environments, providing a fast, inexpensive, and robust way of dealing with otherwise complex and important problem.

We suggest that further research should concentrate on larger, and more sophisticated interior environments, perhaps with multiple pick-up points. In the future, we plan on implementing the findings described in this manuscript in real-life environments. Our solution does not solve the problem of malevolent intent from human participants, like purposefully blocking the robots’ movement or sensors. As such, further research could tackle the problem of automatically rating the quality, and completeness of the constructed influence map and stopping movement if the values of the aforementioned parameters are deemed too low. Splitting the influence sources into natural and artificial could also be a potential research direction, as it could allow for prioritization of human traffic over other robots, and further improve the safety of workers. Another potential research direction would be utilizing UAVs instead of ground-based vehicles, perhaps with the combination of a state-of-the-art control algorithm, which takes into account stochastic disturbances [51,52].

## Data Availability

The original contributions presented in the study are included in the article, further inquiries can be directed to the corresponding author.

## Abbreviations

The following abbreviations are used in this manuscript:

GDC	Game Developers Conference
UAV	Uncrewed Aerial Vehicle
IM	Influence map
PF	Potential field
AI	Artificial intelligence
